# Computer-Aided Design of Molecularly Imprinted Polymers for Simultaneous Detection of Clenbuterol and Its Metabolites

**DOI:** 10.3390/polym11010017

**Published:** 2018-12-23

**Authors:** Bingcheng Zhang, Xin Fan, Dayun Zhao

**Affiliations:** 1 Department of Food Science and Technology, School of Agriculture and Biology, Shanghai Jiao Tong University, Shanghai 200240, China; zhangbingcheng@sjtu.edu.cn (B.Z.); fanxin231400@vip.qq.com (X.F.); 2 Bor S. Luh Food Safety Research Center, Shanghai Jiao Tong University, 800 Dongchuan Road, Shanghai 200240, China

**Keywords:** clenbuterol, metabolites, molecularly imprinted polymer, computer simulations, density functional theory

## Abstract

Molecular imprinting technology (MIT) offers an effective technique for efficient separation and enrichment of specific analytes from complicated matrices and has been used for illicit veterinary drug detectionin recent years due to its high selectivity, good chemical stability, and simple preparation. The development of in silico-based approaches has enabled the simulation of molecularly imprinted polymers (MIPs) to facilitate the selection of imprinting conditions such as template, functional monomer, and the best suitable solvent. In this work, using density functional theory (DFT), the molecularly imprinted polymers of clenbuterol and its metabolites were designed by computer-aided at B3LYP/6-31 + G (d, p) level. Screening molecular imprinting components such as functional monomers, cross-linkers, and solvents has been achieved in the computational simulation considerations. The simulation results showed that methacrylic acid (MAA) is the best functional monomer; the optimal imprinting ratio for both clenbuterol (CLB) and its dummy template molecule of phenylephrine (PE) to functional monomer is 1:3, while the optimal imprinting ratio for the two dummy template molecules of CLB’s metabolites is 1:5. Choosin gethyleneglycol dimethacrylate (EDGMA) as a crosslinker and aprotic solvents could increase the selectivity of the molecularly imprinted system. Atoms in Molecules (AIM) topology analysis was applied to investigate the template-monomer complexes bonding situation and helped to explain the nature of the reaction in the imprinting process. These theoretical predictions were also verified by the experimental results and found to be in good agreement with the computational results. The computer-simulated imprinting process compensates for the lack of clarity in the mechanism of the molecular imprinting process, and provides an important reference and direction for developing better recognition pattern towards CLB and its metabolite analytes in swine urine samples at the same time.

## 1. Introduction

Clenbuterol (C_12_H_18_CL_2_N_2_O, CLB), which is used in human and veterinary medicine as a therapeutic drug for the pulmonary disease, is a synthetic β2-adrenoceptor agonist [1]. However, it has been often illicitly abused as a “lean meat agent” in the feed for pig and cattle to improve growth rate, and enhance lean meat-to-fat ratio. More and more investigations have demonstrated that clenbuterol is a medium cumulative drug and residues build in animals, which can lead to symptoms such as muscle chatter, palpitation, trembling, headache, nausea, and vomiting after human consumption of meat products. It is especially harmful to patients with diseases such as hypertension, heart disease, hyperthyroidism, and prostatic hypertrophy [2]. Although there have been no major food safety incidents under the supervision of the government in recent years, according to online public opinion surveys, the illegal use of “lean meat agent” has always been one of the food safety issues that people are most concerned about [3].

Current detection methods for clenbuterol mainly include enzyme-linked immunoassay (ELISA) [4], gas chromatography coupled with mass spectrometry (GC-MS) [5], high performance liquid chromatography (HPLC) [6], liquid chromatography–mass spectroscopy (LC-MS) [7], surface molecularly imprinted polymers [8], electrochemical analysis [9], capillary electro-phoresis [10], and fluorescence biosensor [11]. ELISA is a commonly used technique in rapid detection, but there is a problem of false positives in practical use [12]. GC-MS, HPLC and LC-MS methods can accurately detect clenbuterol, however, there are disadvantages such as long detection cycle and many operation procedures. At the same time, these methods are time consuming and labor intensive, as well as expensive equipment are needed, which are not likely to be rapid, sensitive and appropriate detection for routine monitoring [13].

Compared with the above detection methods, molecular imprinting technology (MIT) enjoys a unique advantage in rapid detection. This yields MIT a wide range of applications infood analysis [14,15,16]. MIT is a process in which a target molecule is used as a template to prepare a polymer matrix, which can selectively rebind the template molecules from a mixture of closely related compounds [17,18]. Because its recognition process is similar to the relationship between enzyme and substrate, molecularly imprinted polymer (MIP) is also called "artificial antibody" [19]. Since Mosbach and co-workers [20] reported on the results of the preparation of MIP of theophylline in the non-covalent approach, MIT technology has gained more and more attention. Recently, with the rapid development of in silico simulation, it is a hot topic in current research to assist in the rational design of MIPs system through computational chemistry [21] and computer simulation plays an important role in determining the optimal functional monomer, optimizing the synthesis conditions of the imprinted substance, and elucidating the mechanism of molecular imprinting recognition [22,23,24]. For instance, based on the density functional theory (DFT), Maouche and Mazouzet al. [25,26] adopted quantum chemical calculation to determine the nature of interactions between each analyte and the polypyrrole matrix and the dopamine imprinted polypyrrole sensing layer. It is worth mentioning that recently Terracina et al. [27] developed a novel in silico method for computationally imprinting and characterizing enantioselective binding sites, which makes a new progress in elucidating the mechanism of imprinting enantioselectivity. Besides, there are also multiple experimental methods utilized to guide the rational design of MIPs. For example, pre-polymerization mixtures, changes in spectral properties, thermodynamic properties, and electrochemical parameters before and after interaction of the template with the monomer can be measured by NMR [28], differential scanning fluorometry [29], isothermal titration calorimetry (ITC) [30] and conductivity measurement [31], which enables quantitatively study of binding affinities and unravel the mechanisms underlying molecular interactions.

More recently, different approaches have been used to synthesize MIPs for CLB detection, practical application of this technique, however, is still lacking, and this study would be conducive to fill this gap in the context of the increasing attention of computational chemistry. Although we have previously reported the results of a novel molecularly imprinted sensor array for the detection of CLB and its metabolites [32], our focus, in the past, was on whether MIP-QCM (quarzt crystal microbalance) could be established and the construction of molecular imprinting systems was sloppy. Hence, more thorough investigations into the rational design of MIPs for both CLB and its metabolites are required. In order to achieve this aim, DFT and AIM-based computational and theoretical approaches were applied in this work for describing, predicting and analyzing molecular imprinting systems. CLB, 4-hydroxymandelic acid (HMA) and 4-Aminohippuric acid (AHA) have been selected as template molecules in the past [32]. Wherein, HMA and AHA are alternative template molecules for two metabolites. In this study, phenylephrine (PE) will also be employed as a dummy template molecule for CLB, thus the designed array will be constituted of four sensors based on four high selective molecular imprinted polymers, which can be developed into a robust and cost-effective method suitable for simultaneous detection of CLB and its metabolites.

## 2. Experimental

### 2.1. Selection of Functional Monomer

**Figure 1:**
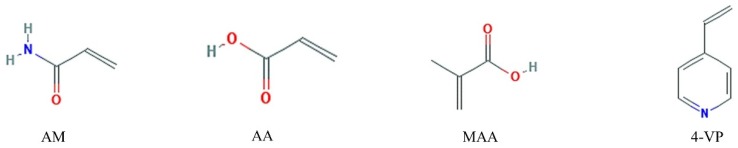
Chemical structures of four functional monomers. AM: acrylamide; AA: acrylic acid; MAA: methacrylic acid; 4-VP: 4-vinylpyridine.

Four functional monomers were employed, namely acidic acrylic acid (AA) and methacrylic acid (MAA), neutral acrylamide (AM) and basic 4-vinylpyridine (4-VP), their molecular structures are shown in Figure 1. The initial conformations of each template molecule and functional monomer were optimized respectively to obtain the energy and the Gibbs free energy of these molecules. The template molecule was then combined with the functional monomer to yield a stable complex conformation with no imaginary frequencies. Depending on the size of the binding energy Δ*E*, the functional monomers that bind to the template molecules were selected. Besides, the Gibbs free energy of molecules could be obtained through Gaussian 09 programs (Gaussian, Inc., Wallingford, CT, USA) [33]. The size of the binding energy can be used to judge the extent of the reaction, while the Gibbs free energy value can be useful to determine the spontaneity of the reaction. Counterpoise method was used to eliminate the basis set superposition error (BSSE), which was proposed by Boys et al. [34].

The BSSE corrected interaction energy and Gibbs free energy are as follows:

(1) $$△E=E_{\mathrm{complex}}-E_{\mathrm{template}}-nE_{\mathrm{monomer}}+\mathrm{BSSE}$$

(2) $$△G=G_{\mathrm{complex}}-G_{\mathrm{template}}-nG_{\mathrm{monomer}}$$

### 2.2. Analysis of Reaction Sites

During the non-covalent approach, the imprint molecules interact, during both the imprinting procedure and the rebinding, with the polymer via non-covalent interactions, e.g., ionic, hydrophobic and hydrogen bonding. The analysis of the site of the imprinting process is pre-judged by the natural population analysis and the molecular electrostatic potential diagram. The natural bond orbital (NBO) analysis allows for calculation of the number of atoms in the molecule, the molecular structure, and the intramolecular or intermolecular hyperconjugation interactions. Using the natural population analysis (i.e., NPA charge) calculated by natural bond orbital theory, the NPA charge transfer between the template molecule and the functional monomer could be judged and the site of action and the magnitude of the force could be predicted. The electrostatic potential diagram maps ESP (electrostatic potential) to an isosurface with an electron density of 0.001 (definition of van der Waals surface by Bader) was used to obtain the electrostatic potential coloring diagram of the surface of the molecule [35,36].

### 2.3. Construction of Template-Monomer Complexes

In order to obtain the optimal conditions for the construction of template-functional monomer-cross-linkercomplexes during imprinting process, not only their charge distributions but also their steric hindrances were taken into account, the ratio of imprinting functional monomers to the target or template molecules was modeled in terms of energy via the strength of the hydrogen bonds under the assumption of generating possible hydrogen bonds until the most stable imprinted complexes were achieved. After obtaining final imprinted complexes, their number of hydrogen bonds, bond length, and bond types were further analyzed. Hydrogen bonds are usually expressed in the form of X–H...Y. X, Y = F, O, N (i.e., atoms of large electronegativity and small radius). O...H is between 0.97 Å (typical O-H length) and 2.6 Å (O–H van der Waals radius) and the bond energy is typically less than 42 KJ/mol. In biological systems, DNA double-helical and protein α-helical and β-structure conformations are extensively hydrogen bonded, in which the length of the hydrogen bond ranges from 2.6 to 3.1 Å [37]. And the nature of hydrogen bond described by Jeffrey is outlined in Table 1 [38].

### 2.4. AIM Topology Analysis

Multiwfn [39] is an extremely powerful program for wavefunction analysis, which is especially good at visual study of real space functions such as electrostatic potential (ESP) and electron localization function (ELF) running on Windows and Linux platform. Bader’s Atoms in Molecules (AIM) topology analysis [40] is the most commonly used and classic method of weak interaction analysis that interprets weak interaction features by the nature of the inter-atomic bond-critical points (BCPs). According to AIM theory, BCP is the most representative point of interatomic interaction [41,42]. Therefore, to a certain extent, the key features of bond can be utilized to understand the properties of BCP. Modeling from the BCP toward the direction of the fastest ascent of the electron density gradient until it encounters the nucleus, its path is called the bond path, which depicts the path of the interaction between atoms.

### 2.5. Cross-Linking Agent Screening

**Figure 2:**
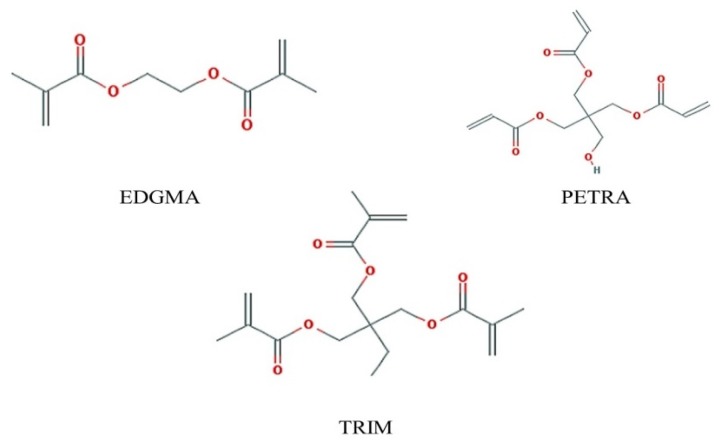
Molecular structures of three cross-linkers. EDGMA: ethyleneglycol dimethacrylate; PETRA: pentaerythritol triacrylate; TRIM: trimethacrylat.

Ethyleneglycol dimethacrylate (EDGMA) is the most commonly used crosslinkers, however, crosslinkers containing 3 or more vinyl groups such as trimethacrylate (TRIM), pentaerythritol triacrylate (PETRA) may make MIP have better column capacity, resolution and selectivity [43,44]. Thus, besides EDGMA, PETRA, and TRIM were also chosen for modeling and analysis. The formula for crosslinker binding energy is

(3) $$△E_{B}=\;E_{C}-E_{T}-E_{M}-E_{\mathrm{CL}}$$

where *E*_C_ stands for the total energy of the cross-linking agent, the template and the functional monomers complex; *E*_T_, *E*_M_, and $E_{\mathrm{CL}}$ represent the single-point energy of monomer, template and cross-linking agent respectively. Their chemical structures are depicted in Figure 2.

### 2.6. Selection of Solvent

PCM model (Polarizable Continuum Model) is one of the most widely used methods since it meets a good compromise between accuracy and computation time, the newest version, G09, includes some improvements on the corresponding codes making PCM calculations more achievable. However, it does not include the calculation of the non-polar part of the solvent effect [45]. The SMD model [46] supported by G09 is by far the best implicit solvent model since the single-point energy given at this time already contains both polar and non-polar contributions. In this study, the solvation energy of each template-monomer-crosslinker system was calculated via the SMD model. According to the polarity of the solvent, five commonly used solvents (acetonitrile (ACN), chloroform (CHF), dimethyl sulfoxide (DMSO), methanol (MeOH), and tetrahydrofuran (THF) were chosen for in silico analysis. The formula for solvation energy is:

(4) $$E_{\mathrm{in}\;\mathrm{solvation}}=E_{\mathrm{in}\;\mathrm{gas}}-E_{\mathrm{in}\;\mathrm{solvent}}$$

### 2.7. Selectivity Examination by Computational Simulation

In order to understand the properties of MIPs at the molecular level, selectivity simulations were performed to examine bias related to the selectivity of previously designed molecularly imprinted polymers by recombination energy size, which shows the adsorption capacity of the imprinted polymer to other template molecules. In this step, the cavity formed was used in the selectivity studies, and the theoretical model of polymer cavity was created on the basis of the most stable prepolymerization complex structure. Subsequently, the template was removed from the complex, and an empty space was proposed as a computer model of the binding site in the polymer matrix. The trial molecules of analogues were inserted into the cavity replacing the template molecule. The binding energy was correlated with the binding capacity of the selected analytes, and the results from the runs were examined to evaluate the empirical binding scores.

### 2.8. Preparation of MIP-QCM and Measurement of Sensors Response

#### 2.8.1. Surface Cleaning of QCM Gold Electrode

Gold microelectrodes on both side surfaces of QCM were polished and cleaned in Piranha solution (30% H_2_O_2_: 98% H_2_SO_4_ 1:3, *v*/*v*) for 10 min. Then, rinsed with deionized water and dried by nitrogen rinsing.

#### 2.8.2. Self-Assembled Monolayer (SAM) of QCM

The QCM was placed into 20 mL of 10 mM solution of 11-mercaptoundecanoic acid in ethanol, kept at 20 °C for 24 h and then was rinsed with ethanol and deionized water.

#### 2.8.3. Preparation of AIBN-QCM

The carboxyl groups were activated by placing the resonator in 10 mL of 10 mM aqueous solution of 2-ethyl-5-phenylisoxazolium-3′-sulfonate for 30 min. The resonator was then transferred into methanol solution of 20 mL of 10 mM AIBN, 0.25 mM DMAP, 1.0 mM DCC and kept at 20 °C for 5 h. The initiator AIBN was then covered on the gold electrodes.

#### 2.8.4. In Situ Preparation of MIPs

2.5 mmol of CLB and 7.5 mmol of MAA, (Alternatively, 2.5 mmol of HMA and 12.5 mmol of MAA; or 2.5 mmol of AHA and 12.5 mmol of MAA) were dissolved in 10 mL acetonitrile. Subsequently, 71 μL of EGDMA was added and the QCM was dipped. The reaction system was purged with nitrogen and then left to polymerize overnight at 65 ± 2 °C for 12 h. Finally, MIP-QCM was slowly rinsed with 5% acetic acid solution to remove template molecules.

#### 2.8.5. MIP-QCM Performance Test

The measuring apparatus for sensor array construction and the collected signal records were performed as our previously established method [32]. The specific parameters of the network analyzer were set as follows: the scan type was set to linear scan; the scan frequency span was 30 kHz; the number of scan points was 12,801 points; the smoothing function was turned on, and the average factor was set to 3. The frequency resolution is 30,000/12,801 = 2.34Hz.

### 2.9. Statistical Analysis

Data were expressed as means ± S.D. and statistical significance was determined using one-way ANOVA, followed by Tukey’s test.

## 3. Results and Discussion

### 3.1. Theoretical Selection of Functional Monomer

Density functional theory (DFT) method in B3LYP level with 6-31*G* (d, p) [47] basis set has been widely applied to obtain the most stable configurations and binding energy for qualitative analysis of the hydrogen bonding-dominant weak interaction in molecular imprinting process [48], because the property of electron cloud deformation could be effectively and accurately predicted in the modeling. However, in the simulation process, it was found that affected by the interference of some strong influence points, the change trends of △*E* and △*G* did not show strong consistency. Diffuse s- and p- functions for non-hydrogen atoms were then added in order to obtain a higher accuracy. It has proved that the diffuse function does make the simulation results more refined and high consistency of △E and △*G* was achieved. The correlation coefficient between △*E* and △*G* reaches 0.955. Therefore, 6-31 + *G* (d, p) [49] was chosen as the basis set for the computational simulation.

**Figure 3:**
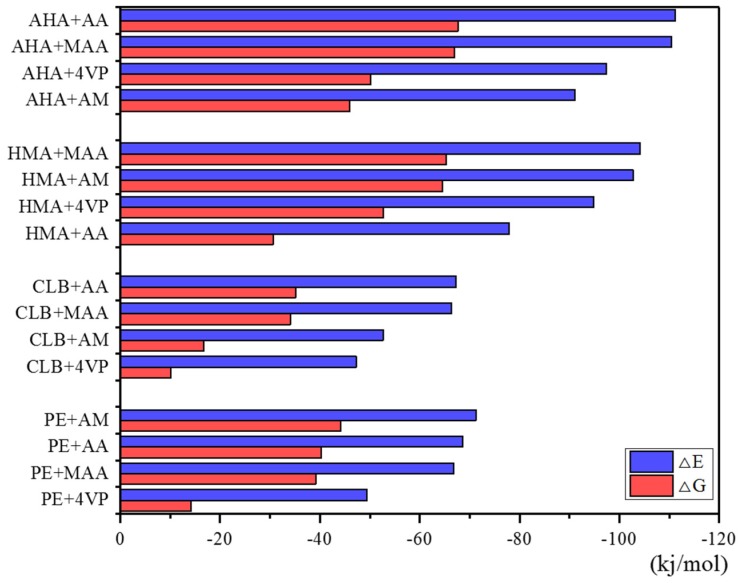
△*E* and △*G* of complexes with imprinting (monomer/template) ratio = 1. Note: Gibbs free energy of molecules was obtained through Gaussian 09 programs, and the Δ*E* and Δ*E* calculation was illustrated in the section of the Experimental. 4-aminohippuric acid, AHA; acrylic acid, AA; methacrylic acid, MAA; 4-vinylpyridine, 4-VP; 4-hydroxymandelic acid, HMA; *N*-dicyclohexylcarbodiimide; clenbuterol, CLB; pentaerythritol triacrylate.

In the meantime, it can be seen from the Figure 3 that two acidic functional monomers, AA and MAA, showed stronger binding capacity with clenbuterol and its metabolites compared to the neutral monomer AM and the basic monomer 4-VP. Considering the poor performance of the combination between AA and HMA, and MAA has been more commonly used in molecular imprinting, MAA was chosen as the functional monomer. 

### 3.2. Theoretical Selection of Template Molecules and Determination of Functional Monomer Site of Action

Computing by molecular self-assembly, the template molecule and the selected functional monomer MAA are supposed to form a stable complex configuration. The spatial conformation of the complex, the sites of hydrogen bonding and the number of hydrogen bonds, all of these will all have a direct impact on the final imprinting effect. The construction of the initial position and conformation of the final stable complexes were calculated with the assistance of NPA charge and molecular electrostatic potential (MEP) electrostatic potential diagram in order to find out the possible coordination modes of the template compound with functional monomers. 

**Figure 4:**
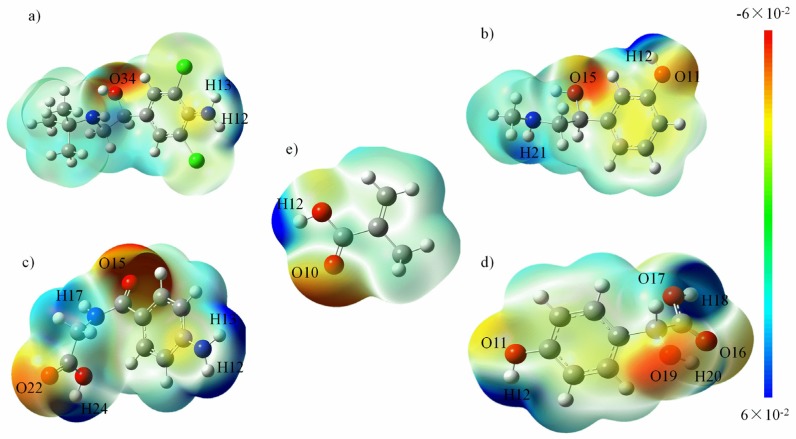
Electrostatic potentials on the molecular surfaces of (**a**) CLB, (**b**) PE, (**c**) AHA molecules, (**d**) HMA and (**e**) MAA. Note: molecular electrostatic potential (MEP) map and the natural population analysis (NPA) charge of each atom were applied to analyze the active sites and to construct the template-monomer complex, please refer to the section of the Experimental for details.

The molecular electrostatic potential represents the attraction between the molecule and a proton, which is useful in rationalizing the interactions between molecules and molecular recognition processes. A map of the electrostatic potential onto the molecular surface of four templates and functional monomer MAA is shown in Figure 4. According to the distribution of the electron cloud, the active sites can be directly predicted. On the basis of comprehensive consideration of the spatial conformation, the MEP map and the NPA charge of each atom were applied to analyze the active sites and to construct the template-monomer complex. As indicated in Figure 4e, the proton donor of MAA is H12 and its proton acceptor is O10; the proton donors of CLB in Figure 4a are H12, H13 and its proton acceptor is O34, N11, N19; while the proton donors of PE in Figure 4b are H12, H21 and its proton acceptors are O11, O15, N20. In Figure 4c,d, the template molecules are AHA and HMA respectively. Compared with CLB and PE, these two template molecules hold more active sites and their carboxyl and carbonyl groups have a higher reactivity. Proton donors of HMA are H12, H18, H20 and its proton acceptors are O11, O16, O17, O19; proton donors of AHA are H12, H13, H17, H24 and its proton acceptors are O15, O22, N11, N16 respectively. In addition, the NPA charges of the active sites of each template molecules are listed in the Table 2, which is consistent with the MEP distribution analysis.

### 3.3. Formation of the Template-Monomer Complexes

**Figure 5:**
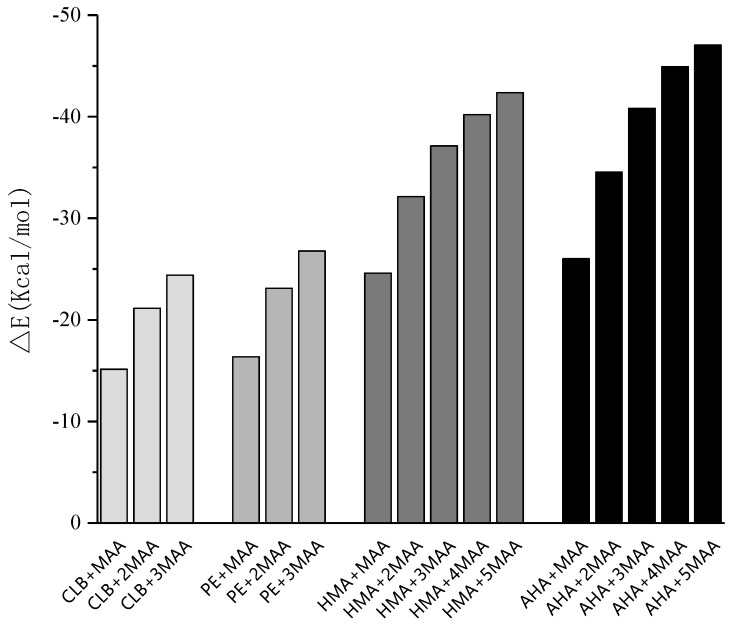
Binding energies of each template-monomer complexes at different molar ratios.

The hydrogen bonding interactions between the active interaction sites play an important role in the formation of MIPs. Suitable molar ratio between template molecule and functional monomer will enable the prepared MIP with desired recognition property. In the present study, the different molar ratios were chosen for simulation. For each imprinting system, simulations were started from 1:1 molar ratio until the most stable conformations were reached. As the imprinting ratio increased, the binding energies were gradually reduced, and the complexes became more stable. Exceeding the upper limit of the optimum ratio, undesired hydrogen bonds could be formed via the non-characteristic bonding points between the monomers, which might lead to lower the selectivity of the synthesized polymers. The detailed binding energies changing trend is shown in Figure 5. 

**Figure 6:**
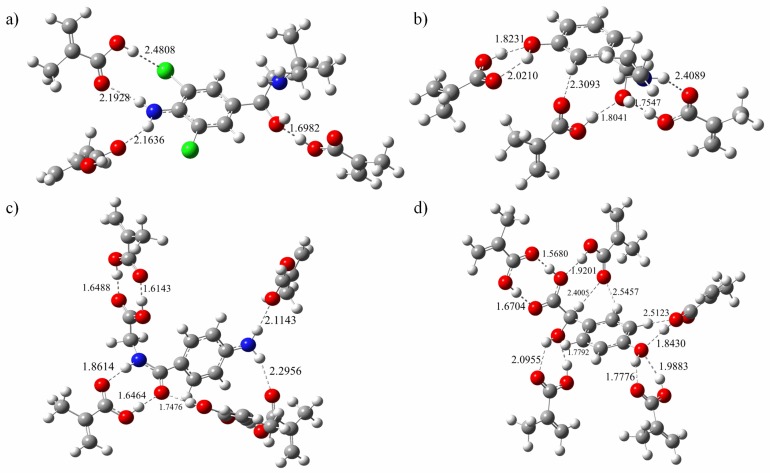
Conformation of template-monomer complexes with the optimized ratios. (**a**) CLB + 3MAA, (**b**) PE + 3MAA, (**c**) AHA + 5MAA and (**d**) HMA + 5MA.

The final configuration of the complex was depicted in the Figure 6. The optimal conditions were obtained, which were as follows: The molar ratio of template to functional monomer for CLB and PE is 3, and the molar ratio of template to functional monomer for AHA and HMA is 5. Owing to more active molecular sites, both AHA and HMA template molecules have a larger molar ratio of template to functional monomer than CLB and PE, which requires more monomer to form a more stable conformation of the complex.

A quantitative analysis of the formation of hydrogen bond networks is illustrated in Table 3, all formed hydrogen bond lengths were scaled from 1.60243 to 2.47659 Å, just between the range of the general O–H single bond and van der Waals radius. The mean hydrogen bond length of the imprinted molecule complexes was 2.26305, 2.06338, 1.76894, and 2.01016 Å for the template CLB, PE, AHA, and HMA, respectively. It can be seen that molecular imprinted complex constructed from template AHA has the lowest binding energy, which is not only related to the high molar ratio of template to functional monomer, but also to the existence of formed multiple hydrogen bonds in the complex. Linked by the double H bond, two adjacent molecules are approximately coplanar, which interaction pattern can improve the stability of the complex. Similarly, for molecularly imprinted complex formed with template CLB, the number of hydrogen bonds generated is relatively small and the bond length is relatively long due to less active sites on the guest molecules and relatively weaker activity as well.

### 3.4. AIM Topology Analysis

Koch et al. [50] and Lipkowski et al. [51] proposed eight general topological criteria for existence of HB interactions, of which the electron density of ρ(r) (density of all electrons) and ▽^2^ρ(r) (Laplacian of electron density) should be within the ranges of 0.002~0.035 a.u. and 0.024~0.139 a.u., respectively. With respect to the electron density characteristics obtained for the complexes studied, Rozas et al. [52] suggest that these criteria can be used to characterize HBs, i.e., when▽^2^ρ(r) > 0, H(r) > 0, the formation of the electrostatic interaction between the molecules formed weak hydrogen bonds; when▽^2^ρ(r) > 0 and *H*(r) < 0, there is a moderate hydrogen bond between molecules; when▽^2^ρ(r) < 0, *H*(r) < 0, there is a strong interaction between molecules, and most of them are covalent. The resulting calculated properties of the electron density ρ(r) are shown in Table 3. The topological analysis of the *V*(r) (electrostatic potential) is the resultant at each point r, which is the net electrostatic effect produced at the point r by both the electrons and nuclei of the molecule. A positive (negative) value reveals that the electrostatic potential at r is dominated by the charge of the nucleus (electron). The results of the weak interaction between templates and monomers analyzed by Multiwfn are also shown in Table 4.

For adduct CLB + 3MAA, the maximum value of ρ(r) at BCP in hydrogen bond is 0.043535397 a.u. and the minimum is 0.01507693 a.u. The range of ▽^2^ρ(r) is within 0.038173768–0.127935635 a.u. The ρ(r) at BCP-O35 exceeds 0.035 a.u., suggesting that the bond formed at the BCP has a strong large hydrogen bond and a significantly shorter bond length (1.69824 Å). The average hydrogen bond energy of CLB + 3 MAA is 4.89 Kcal/mol.

For adduct PE + 3MAA, the maximum value of ρ(r) at the BCP in the hydrogen bond is 0.03996 a.u. and the minimum is 0.00975 a.u. The range of ▽^2^ρ(r) value is within 0.03516–0.10997 a.u. The ρ(r) of BCP at O23 is over 0.035 a.u., indicating that the hydrogen bond strength is stronger than other hydrogen bonds and the hydrogen bond length is also short (1.75467 Å). The average hydrogen bond energy of PE +3 MAA is 5.89 Kcal/mol, which is a reasonable value as well.

The calculated results show that for the two adducts of AHA + 5MAA and HMA + 5MAA, there are 4 and 5 BCPs with large values of ρ(r) respectively. These sites are formed by the template molecule with a carboxyl group, a carbonyl group and an alcoholic hydroxyl group bonded to the carboxyl group of the monomer MAA. Due to the simultaneous donation of double hydrogen bonds, it can facilitate the tight association of the auxiliary and substrate, and thus appears to be a particularly effective method for polymer synthesis.

### 3.5. Theoretical Selection of Crosslinker

**Figure 7:**
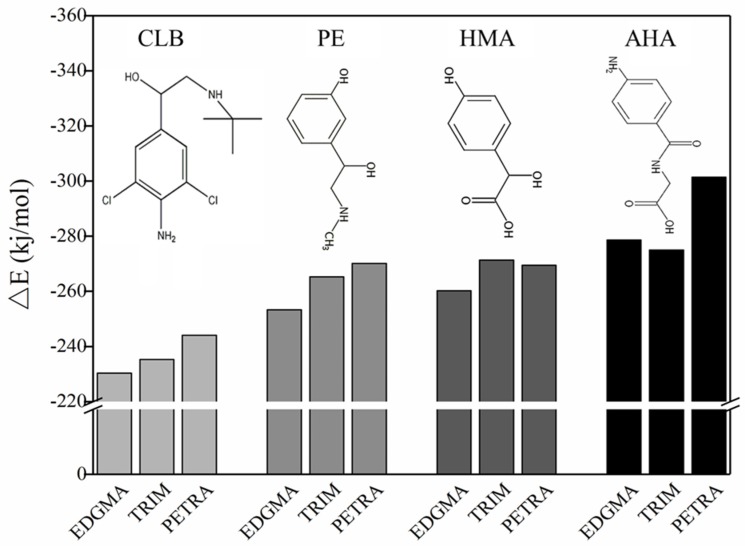
△*E* of cross-linking agents (EDGMA, TRIM, and PETRA) with template-monomer complexes.

In order to make the imprinting process more effective, it is hoped that the functional residues derived from the functional monomers can be uniformly distributed in the entire cross-linked networks. The main function of the crosslinking agent in the entire imprinting process is to copolymerize with the functional monomer and to fix the three-dimensional structure of the monomer-template complex in space. In prepolymerization and copolymerization, the crosslinking agent may complex with both the functional monomer and the template molecule via hydrogen bonding or electrostatic interactions. The formation of such interference complexes is difficult to experimentally define. With the help of computational simulation and calculation, some of the undesirable factors could be avoided. As a result (Figure 7), for CLB, PE, and HMA templates, when EDGMA was used as the crosslinking agent, the interaction between the template molecule and the crosslinker was minimal, that is, the crosslinker has minimal interference with the imprinting process. Compared with crosslinker TRIM or PETRA, EDGMA can reduce unwanted interaction energy by about 3% to 7% for CLB, PE and HMA. For AHA, EDGMA was slightly less effective than TRIM, but it was not much different. Both EDGMA and TRIM had significantly better results than PETRA. EDGMA is a widely used crosslinker with a chemical structure similar to that of MAA. When the random copolymerization of MAA and EDGMA occurs, the product obtained by the crosslinking agent EDGMA and the functional monomer MAA can form a uniform distribution of carboxylic acid groups. In addition, given the limited advantages of TRIM over EDGMA in AHA composites and consistency in practice, the cross-linker was chosen as EDGMA.

### 3.6. Theoretical Selection of Solvent

In the molecularly imprinted polymer synthesis process, the solvent is also known as "porogen" in addition to playing the role of dissolving the polymerization reagents. This is because the solvent can provide a porous structure for the imprinted molecular polymer to increase the speed of bonding the template molecule during recognition. For non-covalent imprinting, the choice of solvent has a direct impact on the formation of non-covalent adducts between the monomer and the template and its imprinting effect.

**Figure 8:**
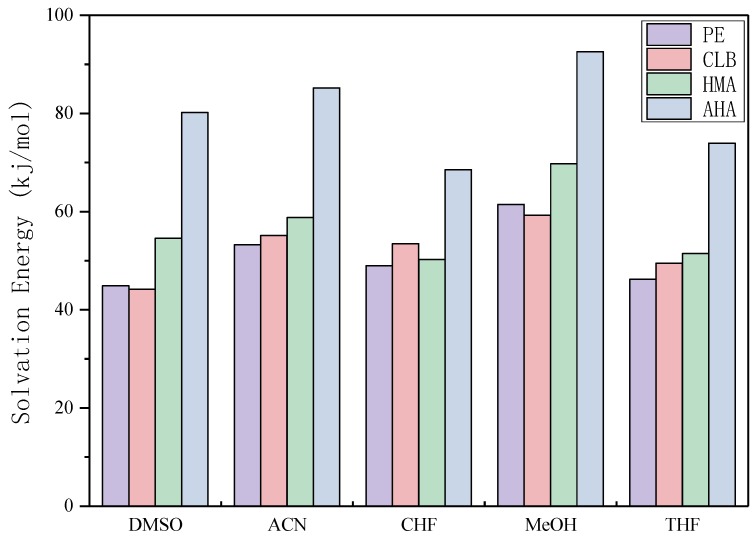
Solvation energy of each template molecules in each chosen solvent (dimethyl sulfoxide, DMSO, acetonitrile, ACN, chloroform, CHF, methanol, MeOH, and tetrahydrofuran, THF).

As can be seen from Figure 8, CHF and THF display lower solvation energy overall and therefore their imprint effect is limited, while MeOH show the strongest effect with each template molecule. The strong interaction between the template molecule and the solvent shields the molecular interaction sites and weakens the interaction with the MAA, making the molecular recognition effect of the imprinted polymer relatively poor. Previously, ACN was used in our laboratory and the solvation energy of the template molecules was relatively high. If CHF and THF were used instead, the specificity of the imprinted polymer could be further improved. However, in practice, it is also necessary to consider the solubility of the template molecule and the functional monomer in the solvent and the porosity of the solvent. Although there are still some realistic factors that need to be coordinated, this calculation result can provide an idea for the direction of improvement.

### 3.7. Selectivity Simulation

**Figure 9:**
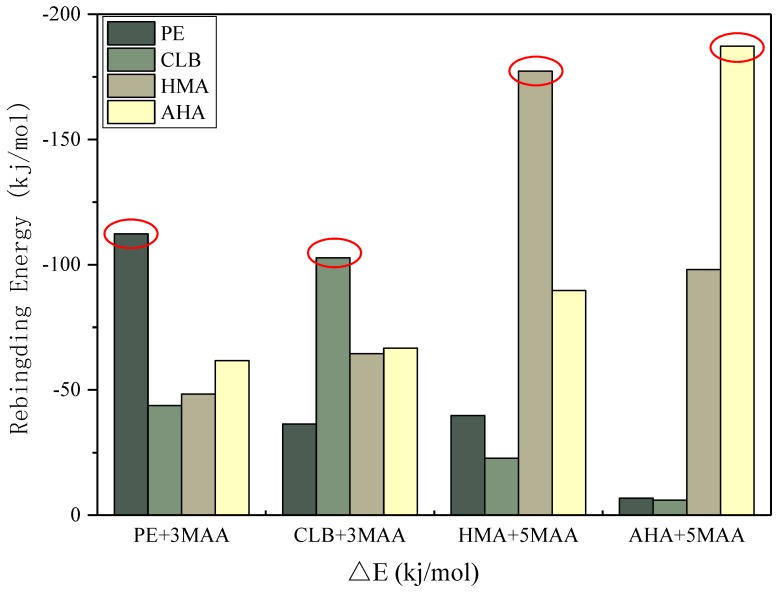
Selectivity test through the rebinding energies of the complexes.

From the results of selective simulation, each single imprinted polymer shows the strongest binding energy for its corresponding target molecule, which reflects the selectivity of molecularly imprinted polymer. In the Figure 9, the binding energy of two guest molecules, AHA and HMA, is greater than that of CLB and PE, because the molar ratio of template to functional monomer of CLB and PE is 3, while for AHA and HMAs, the ratio is 5. This is consistent with the judgment in the previous subsection that both AHA and HMA molecules have more active sites. 

As indicated in Figure 9, each designed imprinted system exhibits a higher specificity for its target molecule, however, it is worthy to be pointed out that in practical applications there are rather few false positive responses depending on the individual molecularly imprinted polymer. Because even a single molecularly imprinted sensor produces a higher response signal, this does not prove to be caused by the target molecule to be captured. In terms of our approach, simultaneous detection of CLB and its metabolites may reduce false positives, because the data measured from the sample is no longer a single dimension of CLB content, but rather the simultaneous detection of CLB together with its metabolites in the pig urine sample, which results in an increase in the data dimension. The data will be then processed by statistical or intelligent algorithms, so that the analysis results for the sample to be tested are more accurate. The selectivity simulation described in this article makes some predictions on the possible situations and provides the forecast and theoretical support for the interpretation of the actual detection.

### 3.8. Experimental Verification

**Figure 10:**
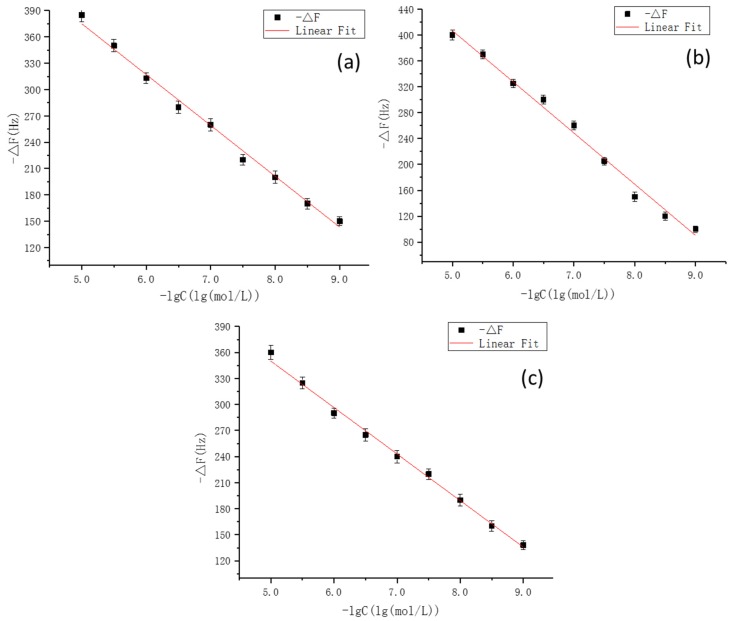
The corresponding frequency shifts of the quarzt crystal microbalance (QCM) sensors for CLB template (**a**), HMA template (**b**), and AHA template (**c**). Note: the synthesis of molecularly imprinted polymers, (MIPs) for each template molecules on gold electrode surface of the QCM sensors, and the construction of the apparatus as well with the measuring method were described in our previously established method [32].

**Figure 11:**
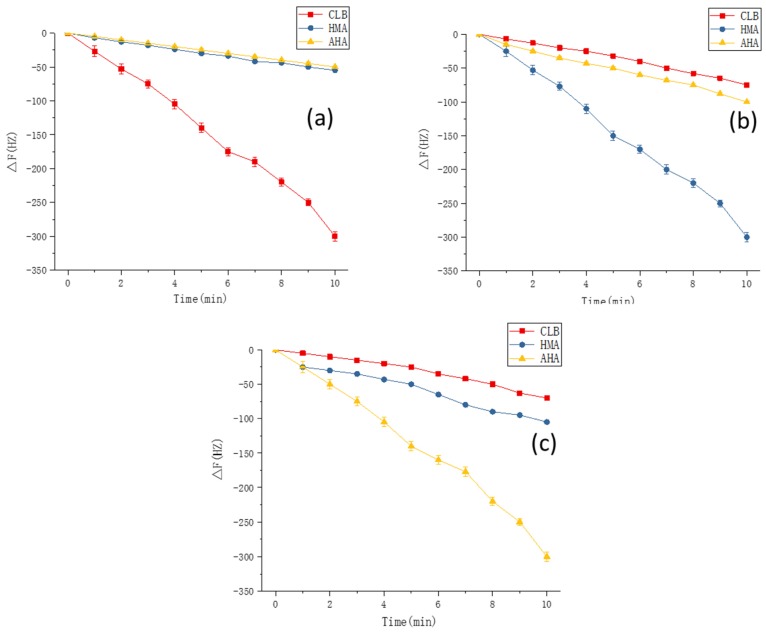
The responses of the MIP-coated QCM sensors with each template solutions over the time. (**a**)CLB-MIP-QCM, (**b**) HMA-MIP-QCM and (**c**) AHA-MIP-QCM. Note: the measuring apparatus for sensor array construction and the collected signal records were performed as our previously established method [32].

Finally, on the basis of the optimal experimental conditions obtained by the simulation, MIPs were synthesized on gold electrode surface of the QCM sensors. To demonstrate the applicability of this method, the fully integrated sensor array was applied to detect CLB and its two metabolites. Standard curves for CLB sensor, AHA sensor, and HMA sensors were plotted and illustrated in Figure 10. The R^2^ values of the three sensors reached 0.9935, 0.9927, and 0.9918, respectively. The 1 × 10^−8^ M Clenbuterol solution was equivalent to a mass concentration of about 3 μg/L, and the selectivity of the sensor was also investigated. As can be seen from the Figure 11, each sensor could specifically recognize the target molecules and the respective signal responses toward the target solutions were 3 times larger than the response values toward non-target solutions (For CLB-MIP-QCM, AHA, and HMA ethanol solutions are non-target solutions; for AHA-MIP-QCM, CLB and HMA ethanol solutions are non-target solutions; for HMA-MIP-QCM, CLB and AHA ethanol solutions are non-target solutions). The experimental results therefore verified the theoretical predictions and found to be in good agreement revealing specific affinity of the prepared MIPs to each target analytes.

However, it is worth noting that, with regard to the real sample determination under the harsh environmental conditions, unknown factors, which may influence the combining capability of the MIPs, should be involved to modify the simulation model and to improve its applicability. In addition, in the future, we are also interested in expanding our screening approach to more complex analyte mixtures (i.e., CLB and its metabolite analytes present in swine urine samples simultaneously). Nevertheless, the method of computationally imprinting enantioselective binding sites allows for greater understanding of the mechanisms underlying MIP binding, and the proposed method provides a promising platform for fabricating simple, fast, and economical sensing system to detect trace amounts of contaminants in food samples.

## 4. Conclusions

In the study, we elucidated the imprinted nature and the interaction mechanization through in silico analysis. Our research covered the geometry optimization, the bonding situation, and the binding energies of selected functional monomers with different proportions to target template molecules in different solvents. The theoretical results showed that MAA was the best functional monomer and EDGMA was the proper cross-linking agent, the optimal imprinting molar ratio of template to functional monomer were 1:3 for both CLB and its dummy template molecule PE, and the ratio were 1:5 for the two dummy template molecules of CLB’s metabolites, respectively. In addition, the predicted recognition of the template molecules towards selected functional monomers in the proper cross-linking agent provided a powerful tool for prediction of the selective biding capability to the synthesized MIPs in the actual tested sample. This study indicates that the experimental and calculated results are consistent, which could provide theoretical references for the preparation of the MIPs, which also revealed that in silico analysis of the properties of molecularly imprinted polymer systems will ultimately allow for the fabrication of more sensitive and selective materials.

## Figures and Tables

**Table 1:** Hydrogen bonding categories following the classification of Jeffrey [38].

Type	Strong	Moderate	Weak
interaction type	strongly covalent	mostly electrostatic	electrostat/dispers
length of H…A (Å)	1.2–1.5	1.5–2.2	>2.2
length of X…A (Å)	2.2–2.5	2.5–3.2	>3.2

**Table 2:** NPA charges and reactive sites of each template molecules.

Template	Reactive Site	NPA Charge	Template	Reactive Site	NPA Charge
CLB	O34	−0.781	PE	O11	−0.713
	H12	0.431		O15	−0.790
	H13	0.432		H12	0.510
	N11	−0.858		H21	0.399
	N19	−0.745		N20	−0.743
HMA	O11	−0.709	AHA	O15	−0.626
	O16	−0.621		O22	−0.607
	O17	−0.711		H12	0.420
	O19	−0.772		H13	0.420
	H12	0.510		H17	0.439
	H18	0.524		H24	0.523
	H20	0.522		N11	−0.861
				N16	−0.677

**Table 3:** Calculated hydrogen-bond properties and distances of the monomer-template complexes.

Complex	△*E* (Kcal/mol)	Type	Length (a.u.)	Complex	△*E* (Kcal/mol)	Type	Length (a.u.)
CLB + 3MAA	−24.41008997	C–O…H–O	1.69824	PE + 3MAA	−26.767172	C=O…H–N	2.40893
		C–H…O=C	2.68041			C–O…H–O	1.75467
		C=O…H–N	2.19275			C=O…H–O	2.02096
		O–H…CL–C(Ring)	2.48079			O–H…O–C(Ring)	1.82306
HMA + 5MAA	−42.36144579	C=O…H–O	1.67043			C(Ring)–H…O=C	2.30929
		C=O…H–O	1.56804			C–O…H–O	1.80407
		C–O…H–O	1.77916	AHA + 5MAA	−47.047349	C=O…H–O	1.64880
		C=O…H–O	2.09553			C=O…H–O	1.61433
		O–H…O–C(Ring)	1.98828			C=O…H–O	1.74762
		C=O…H–O	1.77762			C=O…H–O	1.74762
		O–H…O–C(ring)	1.84295			C=O…H–O	1.64637
		C(Ring)–H…O=C	2.51227			N–H…O=C	1.86144
		C–O…H–O	1.92005			N–H…O=C	2.11431
		C–H…O=C	2.40054			C=O…H–O	1.77100
		C(Ring)–H…O=C	2.54566				

**Table 4:** Electron and energy densities ofthe monomer-template complexesat the hydrogenBCPs (bond critical points).

Complex	H-bond Length (a.u.)	BCP	ρ(r) (a.u.)	▽^2^ρ(r) (a.u.)	*V*(r) (a.u.)	*H*(r) (a.u.)	Energy Kcal/mol
CLB + 3MAA	1.69824	C–O…H–O	0.043535397	0.127935635	−0.032649256	−0.000332674	−10.24385917
	2.19275	N–H…O=C	0.01507693	0.045642552	−0.011384074	1.32821E-05	−3.571807242
	2.48079	O–H…CL–C(Ring)	0.012694051	0.038173768	−0.007446028	0.001048707	−2.336226571
	2.16356	N–H…O=C	0.013996606	0.048719521	−0.010914976	0.000632452	−3.424625563
PE + 3MAA	2.40893	N–H…O=C	0.009747326	0.03516367	−0.007088061	0.000851428	−2.22391283
	1.75467	C–O…H–O	0.039957715	0.109973591	−0.029138968	−0.000822785	−9.142489513
	2.02096	C=O…H–O	0.023316164	0.069012021	−0.018481353	−0.000614174	−5.798612246
	1.82306	O–H…O–C(Ring)	0.03203572	0.096666388	−0.02385453	0.000156034	−7.484472015
	2.30929	C(Ring)–H…O=C	0.013204826	0.039189977	−0.009323669	0.000236913	−2.925345378
	1.80407	C–O…H–O	0.033958284	0.098802002	−0.024766909	−3.32041E-05	−7.770735288
AHA + 5MAA	1.64880	C=O…H–O	0.049287466	0.139181341	−0.037615209	−0.001409937	−11.8019506
	1.61433	C=O…H–O	0.053882161	0.146874063	−0.042683048	−0.002982266	−13.3920091
	1.74762	C=O…H–O	0.038615681	0.112801746	−0.028256176	−2.78698E-05	−8.865509525
	1.74762	C=O…H–O	0.049922233	0.139027425	−0.03838753	−0.001815337	−12.04426994
	1.64637	C=O…H–O	0.031355728	0.089591298	−0.023029819	−0.000315997	−7.225715144
	1.86144	N–H…O=C	0.010611115	0.039224958	−0.008058956	0.000873642	−2.528535829
	2.11431	N–H…O=C	0.016231044	0.052271763	−0.012528486	0.000269727	−3.930871974
	1.77100	C=O…H–O	0.034097227	0.109044945	−0.024677559	0.001291839	−7.74270143
HMA + 5MAA	1.67043	C=O…H–O	0.046441973	0.134085029	−0.034848649	−0.000663696	−10.9339293
	1.56804	C=O…H–O	0.060896745	0.153953802	−0.05136232	−0.006436935	−16.11517194
	1.77916	C-O…H–O	0.036416615	0.10657766	−0.026942346	−0.000148965	−8.453288886
	2.09553	C=O…H–O	0.020289963	0.061744074	−0.016108194	−0.000336087	−5.054022243
	1.98828	O-H…O–C(Ring)	0.023371537	0.065811757	−0.017801058	−0.000674059	−5.585166537
	1.77762	C=O…H–O	0.038597233	0.110446154	−0.029301292	−0.000844877	−9.193419389
	1.84295	O–H…O–C(Ring)	0.030576225	0.089785381	−0.022241089	0.000102628	−6.97824731
	2.51227	C(Ring)–H…O=C	0.008593919	0.028551998	−0.005604602	0.000766699	−1.758470634
	1.92005	C–O…H–O	0.024679618	0.073704853	−0.018155766	0.000135224	−5.696457753
	2.40054	C–H…O=C	0.011004729	0.034475186	−0.007524568	0.000547114	−2.360868924
	2.54566	C(Ring)–H…O=C	0.007706916	0.027055854	−0.005051907	0.000856028	−1.585059833

## Abbreviations

acrylic acid, AA

acetonitrile, ACN

4-aminohippuric acid, AHA

acrylamide, AM

Azobisisobutyronitrile, AIBN

atoms in molecules, AIM

bond-critical points, BCPs

basis set superposition error, BSSE

chloroform, CHF

clenbuterol, CLB

*N*,*N*-dicyclohexylcarbodiimide, DCC

density functional theory, DFT

4-dimethylaminopyridine, DMAP

dimethyl sulfoxide, DMSO

ethyleneglycoldimethacrylate, EDGMA

electron localization function, ELF

enzyme-linked immunoassay, ELISA

electrostatic potential, ESP

4-hydroxymandelic acid, HMA

isothermal titration calorimetry, ITC

methacrylic acid, MAA

methanol, MeOH

molecular electrostatic potential, MEP

molecularly imprinted polymers, MIPs

molecular imprinting technology, MIT

natural population analysis, NPA

natural bond orbital, NBO

polarizable continuum model, PCM

phenylephrine, PE

pentaerythritoltriacrylate, PETRA

quarzt crystal microbalance, QCM

tetrahydrofuran, THF

trimethacrylate, TRIM

4-vinylpyridine, 4-VP
